# Mediterranean Diet and Health-Related Quality of Life in Two Cohorts of Community-Dwelling Older Adults

**DOI:** 10.1371/journal.pone.0151596

**Published:** 2016-03-23

**Authors:** Raúl F Pérez-Tasigchana, Luz M. León-Muñoz, Esther López-García, José R. Banegas, Fernando Rodríguez-Artalejo, Pilar Guallar-Castillón

**Affiliations:** Department of Preventive Medicine and Public Health, Universidad Autónoma de Madrid/IdiPaz, Madrid, Spain; University of Catanzaro Magna Graecia, ITALY

## Abstract

**Introduction:**

In older adults, the Mediterranean diet is associated with lower risk of chronic diseases, but its association with health-related quality of life (HRQL) is still uncertain. This study assessed the association between the Mediterranean diet and HRQL in 2 prospective cohorts of individuals aged ≥60 years in Spain.

**Methods:**

The UAM-cohort (n = 2376) was selected in 2000/2001 and followed-up through 2003. At baseline, diet was collected with a food frequency questionnaire, which was used to develop an 8-item index of Mediterranean diet (UAM-MDP). The Seniors-ENRICA cohort (n = 1911) was recruited in 2008/2010 and followed-up through 2012. At baseline, a diet history was used to obtain food consumption. Mediterranean diet adherence was measured with the PREDIMED score and the Trichopoulou’s Mediterranean Diet Score (MSD). HRQL was assessed, at baseline and at the end of follow-up, with the physical and mental component summaries (PCS and MCS) of the SF-36 questionnaire in the UAM-cohort, and the SF-12v.2 questionnaire in the Seniors-ENRICA cohort. Analyses were conducted with linear regression, and adjusted for the main confounders including baseline HRQL.

**Results:**

In the UAM-cohort, no significant associations between the UAM-MDP and the PCS or the MCS were found. In the Seniors-ENRICA cohort, a higher PREDIMED score was associated with a slightly better PCS; when compared with the lowest tertile of PREDIMED score, the beta coefficient (95% confidence interval) for PCS was 0.55 (-0.48 to 1.59) in the second tertile, and 1.34 (0.21 to 2.47) in the highest tertile. However, the PREDIMED score was non-significantly associated with a better MCS score. The MSD did not show an association with either the PCS or the MCS.

**Conclusions:**

No clinically relevant association was found between the Mediterranean diet and HRQL in older adults in Spain.

## Introduction

In older adults, several cohort studies have shown that greater adherence to the Mediterranean diet is associated with lower mortality.[1–3] Moreover, the Mediterranean diet has been linked to lower risk of death among the elderly surviving a myocardial infarction.[4] There is also evidence that this dietary pattern is associated with reduced risk of cardiovascular diseases,[5] cancer, and depression,[6;7] although most of these studies were not conducted specifically in older adults.

As the population gets older, health outcomes like health-related quality of life (HRQL) or life satisfaction become progressively more important.[8] Thus, for many older adults who already have one or several chronic diseases, the subjective impact of the improvement or deterioration of their physical or mental health can be as important as a diagnosis of a new medical condition.

However, only a few studies have examined the association of a whole dietary pattern with HRQL in adults. A small substudy of the DASH trial assessed the effect of a "combination diet", which emphasized fruits, vegetables and low-fat dairy products, versus the typical American diet; after 8 weeks of follow-up the 28 participants randomly assigned to the "combination diet" modestly improved their HRQL compared to the control group.[9] In addition, three observational studies have found a slightly better HRQL associated with the Mediterranean diet.[10–12] However, two of these studies were of a cross-sectional design and included individuals over the age of 35. [10;12] And the third one was based on a prospective follow-up of relatively young subjects (mean age around 40) but did not assess HRQL at baseline;[11] thus, the beneficial effect of the Mediterranean diet on HRQL could be partly due to differences in baseline physical and mental health status across categories of adherence to the Mediterranean diet.

To our knowledge, this is the first prospective study to examine the association between the Mediterranean diet and HRQL in older adults; it used data from two cohorts of elderly people with measures of diet at baseline, and measures of HRQL at baseline and at the end of follow-up.

## Methods

### Study design and participants

We analyzed data from two cohorts of community-dwelling individuals aged ≥60 years in Spain. The first one was the UAM-cohort, including 4008 individuals selected in 2000–2001.[13;14] Information was collected at the participants’ homes by personal interview and a physical examination, performed by trained staff. In 2003, an attempt was made to contact the study participants again; contact was successful in 3235 (80.7%) individuals and the information was obtained by a phone interview. The second cohort was the Seniors-ENRICA, which included 2519 individuals recruited in 2008–2010 who underwent a personal interview and a physical exam, and provided blood and urine samples in their households.[15;16] In 2012, 2037 (80.9%) participants were contacted again and reported updated information through a phone interview.

Participants in both cohorts gave written informed consent. Both studies were approved by the Clinical Research Ethics Committee of *La Paz* University Hospital in Madrid.

### Study variables

#### Diet

In the UAM-cohort diet information was obtained by a simplified 14-item food frequency questionnaire, based on a validated instrument, which included standard portions of foods and the following response categories: every day, three to five days per week, one to two days per week, or never.[17;18] This information was used to construct a Mediterranean dietary pattern index (UAM-MDP) comprising 8 items, where individuals were assigned +1 point for each one of the following dietary consumptions: fruit every day, vegetables every day, whole grains every day, vegetable oils every day, and fish at least three days per week. Moderate alcohol consumption, defined as an intake of <30 g/day of alcohol in men and <20 g/day in women, also scored +1. Participants scored -1 point for each one of the following consumptions: red or processed meat every day, and animal fats (butter, lard, etc.) every day. The index ranges from -2 to 6, and a lower score indicates a less healthy diet.

In the Seniors-ENRICA cohort, diet was measured with a computerized and validated diet history developed from that used in the EPIC-cohort in Spain.[19;20] Adherence to the Mediterranean diet was assessed with the PREDIMED score [21] and the Trichopoulou’s Mediterranenan Diet score (MSD).[22] The PREDIMED score of 14 items. Twelve of them with food consumption targets (olive oil as the principal source of fat for cooking, >3 tablespoons of olive oil/day, ≥2 serving/day of vegetables, ≥3 servings/day of fruit, <1 serving/day of red meat, <1 serving/day of butter or margarine, <1 serving/day of sugar-sweetened beverages, ≥1 cup/day of wine, ≥3 servings/week of legumes, ≥3 servings/week of fish, <2 servings/week of commercial pastry, ≥3 servings/week of nuts), and two additional items with targets for consumption habits characteristic of the Mediterranean diet in Spain: preference for white meat over red meat, and ≥2 times/week consumption of dishes with *sofrito* (a tomato sauce with garlic, onion, or leeks sautéed in olive oil). A value of +1 was assigned to each target achieved. A higher score indicates better Mediterranean diet adherence (range 0–14). In the MDS, the intake of vegetables, legumes, fruits and nuts, grains, fish, and a high ratio of monounsaturated/saturated fatty acids are considered beneficial, thus a value of 1 is assigned to a consumption above the sex-specific median in the study sample; in contrast, intake of red meat and poultry, and dairy products is considered detrimental, and a value of 0 is assigned to consumption above the sex-specific median. Moderate alcohol consumption is also considered beneficial: 1 point is assigned to intake of 10–50 g/day in men and 5–25 g/day in women. The range in this index is 0 (lowest) to 9 (highest Mediterranean diet adherence).

#### HRQL

In the UAM-cohort, HRQL was measured with the Spanish version of the SF-36 questionnaire, which has shown good validity and reproducibility in the Spanish population.[23–25] In the Seniors-ENRICA cohort, the Spanish version of the SF-12v2 was used. This is a shortened version of the SF-36 which has also been validated in Spain.[26;27]

Both questionnaires measure eight dimensions of HRQL: physical function, physical role, bodily pain, general health, vitality, social functioning, emotional role and mental health. Each dimension has a score from 0 to 100. Information on the eight health dimensions can be summarized by two global HRQL indicators: the physical component summary (PCS) and the mental component summary (MCS). The PCS and MCS scores are standardized to a national norm with a mean of 50 and a standard deviation of 10; this allows comparisons of the scores for each study participant against the mean score in the Spanish population.[28;29] A higher score in each of the eight dimensions and in the PCS and MCS indicates a higher HRQL.

#### Potential confounders

In the two cohorts, data on a number of potential confounders were collected at baseline. Specifically, information was obtained on age, sex, level of education (primary or lower, secondary, and university studies), and tobacco smoking (never-, current-, and former-smoker). Weight, height and waist circumference were measured by trained staff using standardized techniques with the subject barefoot and wearing light clothing. Body mass index was calculated as weight in kg divided by squared height in m, and grouped into three categories: normal weight (≤25 kg/m^2^), overweight (25–29.9 kg/m^2^), and obesity (≥ 30 kg/m^2^). Abdominal obesity was defined as waist circumference >102 cm in men and >88 cm in women. In the UAM-cohort, a single question was used to obtain leisure time physical activity, which was classified as none, occasional physical activity, and regular physical activity.[30] In the Seniors-ENRICA, activity in leisure time was obtained with the questionnaire developed by the EPIC-cohort in Spain and was expressed in METs h/week.[31] Individuals were also asked about the time spent watching television (hours/week).

In each cohort, blood pressure was measured with validated tensiometers under standardized conditions, and hypertension was defined as systolic pressure ≥140 mmHg, diastolic pressure ≥90 mmHg or use of antihypertensive drugs. In the UAM-cohort, individuals reported whether they had ever been diagnosed by a physician with any of the following diseases: diabetes mellitus, hypercholesterolemia, coronary heart disease, stroke, cancer at any site, and depression requiring drug treatment. In the Seniors-ENRICA cohort, we collected data on the same diseases, though information on diabetes mellitus and hypercholesterolemia was also based on laboratory determinations. Specifically, diabetes mellitus was defined as serum glucose ≥126 mg/dl or use of antidiabetic drugs, and hypercholesterolemia as the total serum cholesterol ≥200 mg/dl or use of lipid-lowering drugs.

### Statistical analysis

From a total of 3235 participants in the UAM-cohort study, 853 were excluded for lack of information on HRQL, and 6 for missing values in other covariates. Thus, the final analyses were performed with 2376 participants. Subjects included in the analyses did not differ significantly from those not included in any sociodemographic or lifestyle-related characteristics, except for the average number of chronic diseases, which was a somewhat higher among the former subjects. For the Seniors-ENRICA cohort, from a total of 2037 participants, 80 were excluded for inadequate information on diet, and 46 for missing values in HRQL. Thus, the final analyses were performed with 1911 participants. In the Seniors-ENRICA cohort, and compared to individuals included in the analyses, those not included were less often males, were slightly older and had more frequent chronic conditions

The association between adherence to the Mediterranean diet and quality of life was summarized with beta coefficients and their 95% confidence interval (CI) obtained from linear regression. In these models, the dependent variable was the PCS or the MCS at the end of follow-up, and the main independent variable was adherence to the Mediterranean diet, modeled in tertiles of the score on the UAM-MDP, the PREDIMED or the MDS. A 3-point difference in the beta coefficient for the component summaries of HRQL was considered as clinically relevant.[26;28] Further analyses were performed using each of the eight subscales of the SF-36 and the SF-12 as the dependent variable. All the regression models were adjusted for the above mentioned confounders and for the appropriate HRQL component summary or subscale at baseline.

We assessed whether the study association varied with baseline HRQL (above or below the median), smoking status, physical activity, and morbidity by comparing models with and without interaction terms using the likelihood ratio test. Morbidity was defined as the presence of any of the following diseases: diabetes, hypercholesterolemia, coronary heart disease, stroke, cancer, and depression.

A two-sided p-value <0.05 was considered statistically significant. Analyses were performed with Stata v.11.

## Results

In the UAM-cohort, those with greater adherence to the Mediterranean diet were more often women, had higher education, performed more physical activity, and more frequently had hypercholesterolemia. In the Seniors-ENRICA cohort, those with higher scores in the PREDIMED and MDS had lower body mass index, and less often suffered from abdominal obesity and diabetes (Table 1).

**Table 1:** Baseline characteristics of the participants in the UAM-cohort and in the Seniors-ENRICA cohort, according to tertiles of adherence to the Mediterranean diet.

	UAM-cohort (n = 2376)			Seniors-ENRICA cohort (n = 1911)					
	UAM-MDP Score			PREDIMED Score			MDS Score		
	Tertile 1	Tertile 3	p	Tertile 1	Tertile 3	p	Tertile 1	Tertile 3	p
**n**	966	656		813	458		1000	465	
**Age,** mean (SD)	70.3 (7.0)	69.9 (6.5)	0.420	68.4 (6.4)	68.5 (5.7)	0.424	68.6 (6.2)	68.1 (5.9)	0.376
**Males,** n (%)	416 (43.1)	305 (46.6)	0.020	443 (54.5)	246 (53.7)	0.000	521 (52.1)	168 (36.3)	0.000
**Educational level,** n (%)									
Primary or less	819 (84.7)	508 (77.5)	0.003	419 (51.5)	232 (50.7)	0.034	533 (53.3)	250 (54.0)	0.979
Secondary studies	81 (8.4)	61 (9.2)		215 (26.4)	120 (26.2)		248 (24.8)	117 (25.3)	
University	66 (6.8)	86 (13.1)		179 (22.0)	106 (23.1)		219 (21.9)	96 (2.7)	
**Tobacco consumption,** n (%)									
Never smoker	628 (64.9)	415 (63.4)	0.046	446 (58.9)	263 (57.4)	0.000	574 (57.4)	304 (65.7)	0.046
Former smoker	239 (24.7)	178 (27.1)		283 (34.8)	162 (35.4)		338 (33.8)	126 (27.2)	
Current smoker	99 (10.3)	61 (9.4)		84 (10.3)	33 (7.2)		88 (8.8)	33 (7.1)	
**Body mass index** kg/m^2^, mean (SD)	29.3 (4.5)	29.0 (4.1)	0.421	28.8 (4.4)	28.1 (4.2)	0.011	28.8 (4.3)	27.9 (4.1)	0.000
**Abdominal obesity,** n (%)	915 (94.6)	616 (94.6)	0.697	494 (60.7)	233 (50.8)	0.003	605 (60.5)	250 (54.0)	0.009
**Leisure time physical activity,** n (%)									
None	407 (42.1)	224 (34.1)	0.011						
Occasional	524 (54.2)	393 (60.0)		-	-		-	-	-
Regular	35 (3.6)	39 (5.8)		-	-		-	-	-
**METs** h/week, mean (SD)	-	-	-	21.0 (15.3)	23.8 (15.8)	0.004	21.4 (15.2)	22.1 (14.6)	0.285
**Time watching TV,** (h/week) mean (SD)	-	-	-	18.4 (11.9)	16.3 (9.3)	0.032	18.0 (11.5)	17.8 (11.0)	0.275
**Total energy intake** kcal/day, mean (SD)	-	-	-	2099 (594)	2065 (524)	0.000	2046 (586)	1977 (540)	0.101
**Morbidity at baseline,** n (%)									
Hypertension	449 (46.4)	324 (49.3)	0.340	546 (67.1)	304 (66.3)	0.882	686 (68.6)	308 (66.5)	0.329
Diabetes	178 (18.4)	139 (21.1)	0.417	151 (18.5)	62 (13.5)	0.000	176 (17.6)	51 (11.0)	0.002
Hypercholesterolemia	251 (25.9)	215 (32.8)	0.001	596 (69.9)	314 (68.5)	0.276	689 (68.9)	336 (72.5)	0.235
Coronary heart disease	56 (5.7)	38 (5.8)	0.994	13 (1.6)	5 (1.9)	0.723	18 (1.8)	5(1.1)	0.192
Stroke	27 (2.8)	19 (2.8)	0.777	6 (0.7)	6 (1.3)	0.437	8 (0.8)	6 (1.3)	0.365
Cancer	9 (0.8)	14 (2.1)	0.055	16 (1.9)	8 (1.7)	0.941	21 (2.1)	6 (1.3)	0.510
Depression	116 (12.0)	56 (8.5)	0.056	68 (8.3)	24 (5.2)	0.038	85 (8.5)	32 (6.9)	0.575

Cut-off points for tertiles of the UAM-MDP score: tertile 1: ≤ 3, tertile 2: 4, tertile: 3 ≥ 5 in men and women; Cut-off points for tertiles of de PREDIMED score: tertil 1: ≤ 7, tertile 2: 8, tertile 3: ≥ 9 in men, and tertile 1: ≤ 6, tertil 2: 7 to 8, tertil 3: ≥9 in women. Cut-off points for tertiles of the MDS: tertile 1; ≤5, tertile 2: 6, tertile 3: ≥ 7 in men, and tertile 1: ≤ 4, tertile 2: 5, and tertile 3: ≥ 6 in women; SD: Standard Deviation; MET: Metabolic Equivalents; TV: television. Abdominal obesity: waist circumference >102 cm in men and >88 cm in women.

At baseline, the adjusted mean scores of the summaries and subscales of quality of life in each cohort were similar among those with the lowest and the highest adherence to the Mediterranean diet; the single exception occurred in the UAM-cohort, where those in the highest tertile of UAM-MDP had a lower score in the bodily pain subscale (Table 2).

**Table 2:** Baseline adjusted mean values for the component summaries and the subscales of the SF-36 in the UAM-cohort and of the SF-12 in the Seniors-ENRICA cohort, according to tertiles of adherence to the Mediterranean diet.

	UAM-cohort (n = 2376)		Seniors-ENRICA cohort (n = 1911)			
	UAM-MDP score		PREDIMED score		MDS	
	Tertile 1	Tertile 3	Tertile 1	Tertile 3	Tertile 1	Tertile 3
**Component summaries**						
**Physical component summary,** mean (SD)	45.5 (10.0)	44.3 (10.1)	45.9 (11.3)	46.2 (10.4)	45.7 (11.4)	46.3 (11.1)
**Mental component summary,** mean (SD)	48.9 (11.5)	49.7 (10.9)	52.2 (10.4)	52.1 (9.9)	52.4 (10.4)	52.5 (10.3)
**HRQL subscales**						
**Physical function,** mean (SD)	71.7 (25.2)	72.1 (25.3)	76.9 (29.9)	78.4 (27.6)	76.2 (30.6)	79.0 (28.2)
**Physical role,** mean (SD)	78.3 (37.8)	75.2 (38.6)	82.9 (26.3)	82.8 (24.5)	82.5 (26.7)	84.0 (24.7)
**Bodily pain,** mean (SD)	72.0 (28.4)	68.7 (29.3)	77.7 (30.4)	79.1 (27.0)	78.3 (29.7)	79.4 (29.4)
**General health,** mean (SD)	56.9 (28.4)	60.1 (29.3)	51.5 (21.9)	51.3 (22.2)	51.3 (22.1)	51.1 (23.5)
**Vitality,** mean (SD)	62.4 (24.1)	63.9 (24.4)	71.1 (25.2)	72.3 (24.6)	71.0 (25.5)	73.0 (24.7)
**Social function,** mean (SD)	81.8 (25.9)	83.3 (25.1)	89.9 (22.7)	89.3 (22.1)	90.3 (22.7)	90.5 (21.2)
**Emotional role,** mean (SD)	86.2 (31.1)	85.7 (31.5)	88.0 (21.1)	87.6 (20.5)	88.3 (21.5)	88.6 (20.6)
**Mental health,** mean (SD)	68.2 (22.6)	70.2 (21.5)	76.8 (20.1)	77.4 (19.7)	77.2 (21.0)	78.1 (19.9)

SD: Standard Deviation; Means adjusted for sex, age, educational level (no formal or primary education, secondary education, university), tobacco consumption (never smokers, former smokers, current smokers), body mass index kg/m^2^ (quartiles), abdominal obesity, hypertension, leisure-time physical activity (none, occasional, regular in the UAM-cohort), METs-h/week (quartiles, in the Seniors-ENRICA cohort), time spent watching TV (quartiles h/week, in the Seniors-ENRICA cohort), total energy intake (quartiles kcal/day, in the Seniors-ENRICA cohort), diabetes, hypercholesterolemia, coronary heart disease, stroke, cancer, and depression.

In the UAM-cohort, the baseline score on the UAM-MDP index showed no association with quality of life at the end of follow-up. When compared with the lowest tertile of UAM-MDP, the beta coefficient (95% CI) for the PCS was 0.15 (-0.59 to 0.91) in tertile 2, and -0.41 (-1.14 to 0.31) in tertile 3; for the MCS, the corresponding figures were -0.02 (-1.16 to 1.10) and 0.26 (-0.83 to 1.36).

In the Seniors-ENRICA cohort, a higher PREDIMED score was linked to slightly better PCS; compared to those in the lowest tertile of PREDIMED, the beta coefficient (95% CI) was 0.55 (-0.48 to 1.59) for tertile 2, and 1.34 (0.21 to 2.47) in tertile 3. By contrast, no link to the MCS was evidenced, and the corresponding figures were -0.25 (-1.31 to 0.80) and 0.56 (-0.58 to 1.71). Neither the MDS was associated with the PCS or with the MCS. When compared with the lowest tertile of the MDS, the beta coefficient (95% CI) for the PCS was -0.44 (-1.53 to 0.65) in tertile 2, and -0.36 (-1.53 to 0.71) in tertile 3; for the MCS, the corresponding figures were 0.61 (-1.51 to 1.72) and 0.60 (-0.50 to 1.70). Results were similar in analyses stratified by sex (Table 3).

**Table 3:** Beta coefficients (95% confidence interval) for the association between tertiles of adherence to the Mediterranean diet and the component summaries of the SF-36 in the UAM-cohort and the SF-12 in the Seniors-ENRICA cohort.

Health-related quality of life	UAM-cohort (n = 2376)			Seniors-ENRICA cohort (n = 1911)					
	UAM-MDP score			PREDIMED score			MDS		
	Tertile 1	Tertile 2	Tertile 3	Tertile 1	Tertile 2	Tertile 3	Tertile 1	Tertile 2	Tertile 3
**Physical component summary**									
Total	Ref.	0.15 (-0.59 to 0.91)	-0.41 (-1.14 to 0.31)	Ref.	0.55 (-0.48 to 1.59)	1.34 (0.21 to 2.47)*	Ref.	-0.44 (-1.53 to 0.65)	-0.36 (-1.53 to 0.71)
Males	Ref.	0.56 (-0.53 to 1.67)	-0.56 (-1.69 to 0.57)	Ref.	0.60 (-0.92 to 2.12)	0.99 (-0.44 to 2.44)	Ref.	-0.51 (-2.01 to 0.99)	-0.61 (-2.21 to 0.99)
Females	Ref.	-0.13 (-1.11 to 0.84)	-0.25 (-1.20 to 0.69)	Ref.	0.45 (-0.98 to 1.89)	1.60 (-0.16 to3.36)	Ref.	-0.40 (-1.99 to 1.18)	-0.21 (-1.71 to 1.29)
**Mental component summary**									
Total	Ref.	-0.02 (-1.16 to 1.10)	0.26 (-0.83 to 1.36)	Ref.	-0.25 (-1.31 to 0.80)	0.56 (-0.58 to 1.71)	Ref.	0.61 (-0.51 to 1.72)	0.60 (-0.50 to 1.70)
Males	Ref.	0.31 (-1.32 to 1.96)	0.05 (-1.59 to 1.70)	Ref.	-0.22 (-1.66 to 1.22)	0.51 (-0.85 to 1.88)	Ref.	0.81 (-0.61 to 2.24)	1.26 (-0.24 to 2.78)
Females	Ref.	-0.15 (-1.66 to 1.34)	0.62 (-0.84 to 2.09)	Ref.	-0.30 (-1.84 to 1.23)	0.36 (-1.51 to 2.25)	Ref.	0.33 (-1.36 to 2.03)	0.003 (-1.59 to 1.60)

*p <0.05.

Model adjusted for sex, age, educational level (no formal or primary education, secondary education, university), tobacco consumption (never smokers, former smokers, current smokers), body mass index kg/m^2^ (quartiles), abdominal obesity, hypertension, leisure-time physical activity (none, occasional, regular in UAM-cohort, and quartiles METs-h/week in Seniors-ENRICA cohort), time spent watching TV (quartiles h/week, Seniors-ENRICA cohort), total energy intake (quartiles kcal/day, Seniors-ENRICA cohort), prevalence of chronic diseases (diabetes, hypercholesterolemia, coronary heart disease, stroke, cancer, and depression), and the appropriate baseline component summary of quality of life.

**Fig 1:**
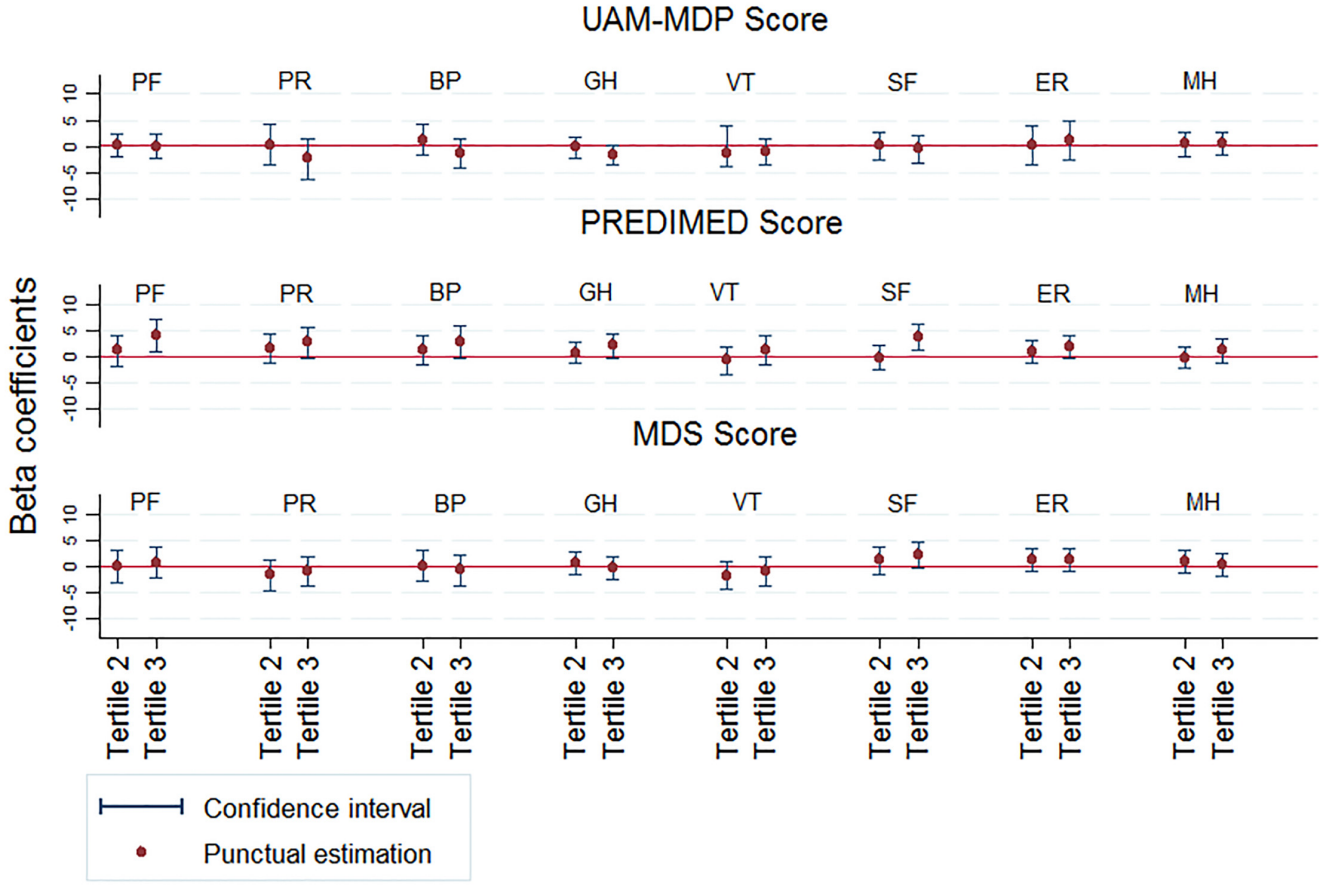
Beta coefficients (95% confidence interval) for the associations between the Mediterranean dietary pattern and the subscales of the SF-36 in the UAM-cohort (2000/2001-2003) and of the SF-12 in the Seniors-ENRICA cohort (2008/2010-13). HRQL subscales: PF: Physical function, PR: Physical role, BP: Bodily pain, GH: General health, VT: Vitality, SF: Social function, ER: Emotional role, MH: Mental health. Model adjusted for sex, age, educational level (no formal or primary education, secondary education, university), tobacco consumption (never smokers, former smokers, current smokers), body mass index kg/m^2^ (quartiles), abdominal obesity, hypertension, leisure-time physical activity (none, occasional, regular in UAM-cohort, and quartiles METs-h/week in Seniors-ENRICA cohort), time spent watching TV (quartiles h/week, Seniors-ENRICA cohort), total energy intake (quartiles kcal/day, Seniors-ENRICA cohort) and prevalence of chronic diseases (diabetes, hypercholesterolemia, coronary heart disease, stroke, cancer, and depression), and the baseline subscale as appropriate.

When the individual quality of life subscales were studied, no association was observed with the score on the UAM-MDP or the MDS. However, those in the highest tertile of PREDIMED reported somewhat better physical and social functioning; the corresponding beta coefficient (95% CI) when tertile 3 was compared with tertile 1 was 3.97 (0.83 to 7.10) and 3.7 (1.13 to 6.32) (Fig 1).

Lastly, no statistically significant interactions were found when the analyses were stratified by baseline HRQL (above or below the median), smoking status, physical activity, or morbidity (data not shown).

## Discussion

In 2 cohorts of community-dwelling older adults in Spain, we found no clinically relevant association between higher adherence to the Mediterranean diet and better HRQL after a few years of follow-up.

Results were generally consistent in both cohorts despite the studies were performed 10 years apart, different instruments to measure HRQL were used, and 3 distinct indexes for measuring the Mediterranean diet were computed. Although the 3 indexes focused on the consumption of fruits, vegetables, meat and fish, the UAM-MDP index was based on normative goals of consumption while the MDS used food consumption medians, which are population-specific. Moreover, the PREDIMED score uses normative goals, emphasizes monounsaturated fat intake, incorporates cooking techniques (e.g., sofrito), assesses consumption of sugary drinks, and includes a greater number of items than the other two indexes.[32]

Our results were in line with those obtained in cross-sectional studies conducted in Spain and in Greece with younger populations. In these studies, a greater adherence to the Mediterranean diet was associated with better HRQL; however, the association was of small magnitude and it did not reach the conventional limits of clinical relevance (>3 points).[10–12] Specifically, in a study in Spain[10], an increase of 5 units in the adherence to the Mediterranean diet (range of 20) was associated with a 0.74-point higher MCS score on the SF-12 in men and a 1.5-point-higher score in women In another study in Spain,^11^ where only the subscales of the SF-36 were computed, a 1-point increase in the MDS was associated with better vitality [beta coefficient (95% CI): 0.50 (0.32–0.68)] and general health [0.44 (0.26–0.52)]. Finally, in a Greek study,[12] a 1-point increase in the MDS was linked to slightly higher score on the PCS [0.15 (0.06–0.24)] and the MCS [0.33(0.18–0.49)] of the SF-36. However, these studies are difficult to compare because of the different components of the dietary indexes used and the lack of normative cut-offs for the intake of a specific food. In fact, in these studies the Mediterranean diet adherence was based on the median intake of the different food groups, which are population-specific.

Several clinical trials have assessed the effect of dietary interventions promoting a healthy diet on the HRQL of some types of patients. Specifically, a cholesterol-lowering diet did not improve mood among hypercholesterolemic patients;[33] a low-fat, high-carbohydrate diet did not improve HRQL among patients with high cardiovascular risk; [34] and a low-fat diet intervention did not modify HRQL in women at high risk of breast cancer.[35] One possible explanation for these results is that, for many people, the prescription of a healthy diet is perceived as an unpleasant experience, which may counterbalance the beneficial health effects of these diets. Thus, efforts must be made to improve the palatability of healthy diets, for instance, by increasing the quality of foods, their affordability, and by promoting cooking skills.

There are several mechanisms by which diet may affect health, including the reduction of traditional cardiovascular risk factors,[36] the decrease of serum markers of inflammation,[37] and improvement of endothelial function.[38] However, most of these effects, including those on lowering blood pressure, cholesterolemia and glycemia, can be asymptomatic, and thus may have a small impact on HRQL. By contrast, a high adherence to the Mediterranena diet index used in this study has previously shown an inverse association with general mortality after 10 years of follow-up in the UAM-cohort.[3] Consequently, regardless of its effects on HRQL, the Mediterranean diet should be promoted among the elderly due to their benefits on other health variables.

Finally, the evidence of the influence of specific food components of the Mediterranean diet on HRQL is still limited. Although several studies have examined the association of consumption of fish,[39,40] fruit and vegetables,[41] and nuts[42]with HRQL, these investigations were mostly cross-sectional or showed inconsistent results.[39,40] In addition, regular yogurt consumption, that is also considered healthy, has not shown an association with improved HRQL in a longitudinal study.[43]

Our study had several strengths. It used data from two independent cohorts, diet and HRQL were measured with validated instruments, and analyses were adjusted for many potential confounders, including HRQL and morbidity at baseline.

This study also had some limitations. Information on HRQL was lacking for 853 subjects in the UAM cohort, and its potential influence on study results is unknown. Also in both cohorts diet was self-reported, which may result in some recall or social desirability bias and the ensuing non-differential misclassification of diet; this and some inaccuracies in other self-reported variables could have led to underestimate the association between the Mediterranean diet and HRQL. Furthermore, in the UAM-cohort the consumption of vegetable oils but not of olive oil was recorded, and olive oil is considered one of the main components of the Mediterranean diet. However, olive oil is by far the oil most frequently used in Spain.[44] In addition, the SF-36 and the SF-12 are generic HRQL questionnaires, and they cannot capture some aspects of perceived health related to diet, such as satisfaction with food-related life,[45] the ability to eat independently, the ability to buy and cook the own food, or food palatability.[8;46] Another potential limitation, which is shared with many cohort studies, is the unverified assumption that the dietary pattern registered at baseline is maintained throughout the follow-up; in fact incident diseases could have led to dietary changes, so that the results might not entirely be due to baseline diet. In addition, in both cohorts the duration of follow-up has been relatively short (median 3.5 years). However, in clinical trials with the Mediterranean diet, improvements in cardiovascular risk factors and in disease incidence and mortality were observed after a few months of follow-up.[47;48] Notwithstanding this, a longer follow-up might be needed to observe a beneficial effect of diet on HRQL. Finally, because the Mediterranean diet is part of an overall lifestyle, a certain residual confounding cannot be ruled out. Also, maintaining a good health throughout the aging process could lead to higher adherence to a Mediterranean diet; thus, despite the longitudinal study design, the observed results could partly reflect reverse causation.

In conclusion, although adherence to the Mediterranean diet has been associated with lower mortality among the elderly in some Mediterranean countries, it does not seem to be associated with a clinically relevant improvement in HRQL. However, since some aspects of the impact of diet on HRQL may not have been captured with the SF-36 and the SF-12 questionnaires, further research should be conducted using comprehensive diet-specific HRQL questionnaires in the elderly.
