# Correction: Mediterranean Diet and Health-Related Quality of Life in Two Cohorts of Community-Dwelling Older Adults

**DOI:** 10.1371/journal.pone.0155171

**Published:** 2016-05-03

**Authors:** Raúl F Pérez-Tasigchana, Luz M. León-Muñoz, Esther López-García, José R. Banegas, Fernando Rodríguez-Artalejo, Pilar Guallar-Castillón

There are errors in the Funding section. The correct funding information is as follows: Baseline data collection of the ENRICA-Seniors cohort was funded by Sanofi-Aventis. Data collection during follow-up was funded by FIS a grant 09/162 (Ministry of Health of Spain). Specific funding for this analysis was obtained from FIS grants PI11/01379 and PI12/1166 (Ministry of Health of Spain, State Secretary of R+D and FEDER/FSE), and from the "Cátedra UAM de Epidemiología y Control del Riesgo Cardiovascular". RFP-T received a grant from the National Government of Ecuador through the National Institution of Higher Education, Science, Technology and Innovation-SENESCYT. The funders had no role in study design, data collection and analysis, decision to publish, or preparation of the manuscript.
